# Environmental regulation, green innovation, and international competitiveness of manufacturing enterprises in China: From the perspective of heterogeneous regulatory tools

**DOI:** 10.1371/journal.pone.0249169

**Published:** 2021-03-30

**Authors:** Yuanhong Hu, Sheng Sun, Yixin Dai

**Affiliations:** 1 College of Business, Shanghai University of Finance and Economics, Shanghai, China; 2 College of Economics, Nanjing University, Nanjing, Jiangsu, China; China University of Mining and Technology, CHINA

## Abstract

Based on combined data from the China Patent Database, China Industrial Enterprise Database, and China Customs Import and Export Database for the period 2004–2010, this study investigates the impact of heterogeneous environmental regulations on the export technological sophistication of manufacturing enterprises. Given deepening international market segmentation of production and the increasing proportion of intermediate trade, and compared with the traditional method based on exports, the export technological sophistication calculated here, based on value-added, is closer to the true level. Since there has been no in-depth comparative study on the relationship between heterogeneous environmental regulation and export technological sophistication, this study fills the gap. The results show that all three regulation types bear a U-shaped impact on export technological sophistication. Command-control regulation exhibits a restraining effect on mixed trade, eastern, and foreign-funded enterprises. Market-incentive regulation promotes processing and mixed trade enterprises as well as domestic and foreign-funded enterprises. Voluntary-participation regulation promotes all enterprises with different trade patterns and ownership. The mechanism analysis shows that command-control and market-participation environmental regulations affect export technological sophistication through the green invention and green utility innovation channels, while, additionally, market-incentive environmental regulation affects export technological sophistication through the green design innovation channel. Considering the environmental governance issues, the policy implications for enhancing the entire industrial chain and enterprises’ export competitiveness are clear. Due to the unclear functions and powers of competent departments and a rigid threshold, command-control regulation is not conducive to cleaner production technology and the promotion of enterprises’ export competitiveness; it should thus be discouraged. Although both market-incentive and voluntary-participation regulations have promoted cleaner production technology and enterprises’ competitiveness significantly, the environmental tax system requires continuous improvement. The government should continue to raise public involvement in environmental protection to enrich the channels and forms of environmental management.

## Introduction

After more than three decades of development, China has achieved rapid export growth through a reliance on low production costs. However, China’s long-standing trade strategy of "two ends outside, massive imports and massive exports" has produced many negative effects. Among the negative consequences are a general lack of competitiveness among enterprises and a reduced quality of goods. The deepening global specialization has not been effective in improving the segments and raising the status of Chinese enterprises from the low end of the global value chain, while the current production pattern with high input costs and high pollution has impacted the domestic environment negatively and caused many social problems. Meanwhile, China’s export trade is under great pressure. Thus, to promote the country’s trade development and to accelerate the transformation of its trade characteristics from "massive imports and massive exports" to "qualified imports and qualified exports", the central government has raised environmental governance to an unprecedented new level by explicitly requiring that major environmental problems should be resolved and pollution prevention and control measures be continuously implemented.

At present, clarifying the relationship between environmental governance and trade upgrading has gradually become a research hotspot in related fields. How does environmental regulation affect enterprises’ export competitiveness? What is the role of enterprises’ green innovation between regulation and competitiveness? To answer the above questions, the investigation of the impact of environmental regulation on enterprises’ export technological sophistication in this study has definite theoretical and practical significance. In the construction of environmental protection and economic growth, governments cannot reach consensus on the trade-off issue between, on the one hand, preserving the ecology of the environment and, on the other, realizing economic growth. The reason is that the introduction of environmental protection policies and measures will "internalize" the cost of environmental pollution and increase the production costs of local manufacturing enterprises. In the trade-off between environmental protection and economic growth, there will be a natural preference for the latter at the expense of the former, and therefore environmental regulation policies cannot be implemented effectively. This study provides theoretical support to relevant policy makers in decisions on ecological environmental governance and high-quality development of foreign trade.

Our study has three marginal contributions to the existing research. First, we incorporate different types of environmental regulations into a unified framework. In the measurement of environmental regulation intensity, previous studies usually choose several indicators in an attempt to build a comprehensive index to reflect and measure the overall level of environmental regulation [1–4]; in some cases, scholars only used some of the tools of environmental regulation in terms of the category of index selection, command-control [2, 5, 6], market-incentive [7–9], voluntary-participation [10, 11] and so forth. However, this approach is not appropriate. Indeed, environmental regulation, as an external constraint mechanism that interferes with the production activities of enterprises, has a relatively broad concept and can be divided into different categories based on different standards. For example, from the perspective of regulatory tools, environmental regulation can be divided into three main types: command-control, market-incentive, and voluntary-participation; there are essential differences between these regulatory tools.

Second, the measurement of export technological sophistication in the existing literature is usually based on exports at country and industrial levels [12–14]. However, the measurement of the export technological sophistication index is not accurate, considering that international market segmentation and intermediate trade proportion continue to improve. Therefore, following [15] and [16], we use the export value-added of manufactured products as a new basis to improve the accuracy of measuring enterprises’ export technological sophistication. This approach will shed more light on the evolving characteristics of Chinese manufacturers’ international competitiveness.

Third, there has been fruitful research on environmental regulation and the complexity of export technology by scholars in their respective fields. For instance [17], examined the correlation between environmental regulation and industrial agglomeration. [18] demonstrated the double threshold effect of external costs on export technological sophistication based on provincial data analysis. [19] empirically tested the impact mechanism of environmental regulation on export quality from the perspective of quality competitiveness. However, no scholars have yet incorporated different types of environmental regulations into a unified framework to conduct comparative analysis on the impact of export technological sophistication.

## Theoretical analysis

### Theoretical analysis

**Fig 1:**
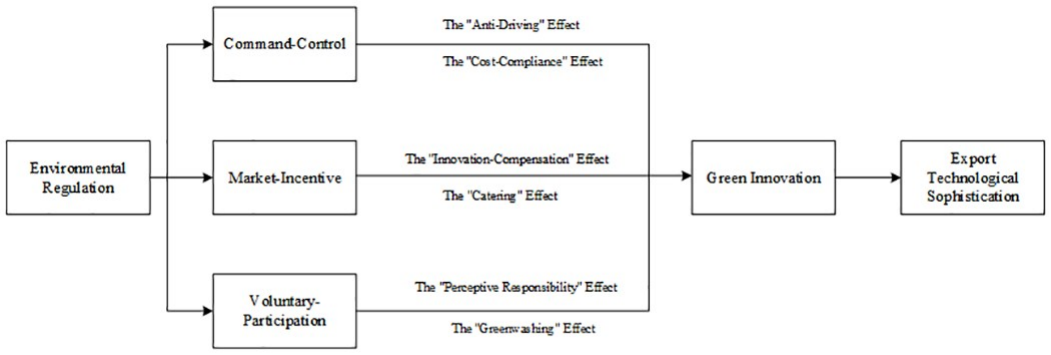
Theoretical mechanism of the impact of environmental regulation on the export technological sophistication of enterprises.

Based on the standard of regulatory tools, we divide China’s environmental regulations into three types: command-control, market-incentive, and voluntary-participation. We analyze the theoretical mechanism of the impact of environmental regulation on export technological sophistication. Many studies have confirmed that an enterprise’s technological innovation, including green innovation, is a crucial factor in driving export technological sophistication [20–23]. Green innovation promotes the international competitiveness of enterprises through cleaner production technology and optimal allocation of green factors. Therefore, the focus in our theoretical analysis is the mediating role of green innovation in the relationship between environmental regulation and export technological sophistication. The theoretical mechanism is shown in Fig 1.

#### The impact of command-control environmental regulation on export technological sophistication

Command-control environmental regulation mainly refers to the kind of policies that are released by an administrative department of government. Regulation is implemented through legislation and regulation rules to determine specific targets and standards of environmental regulation, and enforcement of compliance by the regulated enterprises by way of administrative orders; economic—and even political—penalties are imposed on enterprises that violate the applicable standards and fail to achieve the regulatory goals [24–26]. Depending on the setting, specific means of command-control environmental regulation mainly include environment-related technical and performance standards [27].

Based on a study by [28], we consider two representative enterprises in a certain region, both competitive and driven by profit maximization. When the region begins to implement command-control environmental regulation, Enterprise 1 improves its export competitiveness through green innovation, while Enterprise 2 does not choose green innovation. Without loss of generality, it is assumed that Enterprise 1 and Enterprise 2 produce homogeneous products in quantities *q*_1_ and *q*_2_, respectively; the unit production cost for both enterprises is *z*, and the counter-demand curve for the enterprises is *P* = *m-n*(*q*_1_*+q*_2_), where *P* is the production price of the product, and (*q*_1_*+q*_2_) is the total output. In the process of production, unexpected outputs, such as pollutants, will be produced. When command-control environmental regulation imposes constraints on the enterprises, Enterprise 1 will reduce *s* unit pollutants by means of green innovation. Following the setting in [29], the discharge reduction cost for enterprises is *as*^2^/2, where *a* is the green innovation coefficient, the cost of green innovation is *A*^2^/2, and *A*^*’*^ = *A*^’^(*a*)<0. Enterprise 2 is not subject to regulatory punishment for reducing pollution discharges through green innovation, and faces a penalty cost *X*. The profit functions for Enterprise 1 and Enterprise 2 are respectively:

$$\pi_{1}=[m-n(q_{1}+q_{2})-z]q_{1}-as^{2}/2-A^{2}/2$$ (1)

$$\pi_{2}=[m-n(q_{1}+q_{2})-z]q_{2}-X$$ (2)

$$s.t.\;bq_{1}-s=\bar{E}\;b\in [0,1]$$ (3)

Where *b* is the pollutant discharge coefficient, which is the proportion of pollutant discharge in the product output of the enterprise, *bq*_1_ is the pollutant discharge amount for Enterprise 1, and *E* is the discharge limit specified by the regional environmental regulation. Therefore, the economic significance of constraints lies in the fact that Enterprise 1 must emit less than or equal to the discharge limit set by the command-control environmental regulation. By constructing a Lagrange function, the partial derivatives of the above equations are obtained by taking the partial derivatives of the outputs *q*_1_ and *q*_2_, the discharge reduction *s*, and the Lagrange multiplier *λ*. Algebraic manipulation of the above analytic equations yields the equilibrium outputs *q*_1_ = (*m*-2*asb-z*)/3*n* and *q*_2_ = (*m+asb-z*)/3*n*, and the two profit functions can be subtracted to get.

$$\Delta \pi_{o}=\pi_{1}-\pi_{2}=X-(m+asb-z)asb/3n-as^{2}/2-A^{2}/2$$ (4)

Clearly, from Eq 4, the difference in profit between the enterprises is affected by various factors, such as the regulatory penalty cost *X*, the green innovation level *a*, the discharge reduction cost *as*^2^/2, and the green innovation cost *A*^2^/2. In the case of command-control environmental regulation, only when Δ*π*_*o*_>0 will enterprises pursuing profit maximization have an internal motivation for green innovation. The economic significance of the above mathematical model is that the impact of command-control environmental regulation on exports technological sophistication is bidirectional, and has both potentially positive and negative effects.

On the one hand, command-control environmental regulation has a positive impact on export technological sophistication through the mechanism of "anti-driving" effect. Command-control environmental regulation has strict technical standards that local enterprises must accept and comply with, otherwise they will suffer additional violation costs as the threshold for enterprises are mandatory and obedience of rules and regulations. Based on previous studies, command-control environmental regulation can improve export technological sophistication through three potential channels: First, it strengthens green technological innovation at the production and discharge ends, such as waste discharge reprocessing and waste reuse, thus forming the effect of "pollution command-control progress" [30]. Second, it strengthens green technological innovation in the intermediate stages of production, and reduces the degree of pollution from unit production by improving technology and increasing labor productivity [31, 32]. Third, based on the strong Porter hypothesis, environmental regulation breaks the original production mode of enterprises and transitions them to a new production mode.

On the other hand, command-control environmental regulation also has a negative impact on exports technological sophistication through the mechanism of "compliance cost" effect. When the government tightens its command-control and punishment on enterprises, it will raise the cost of environmental governance, compliance cost, to meet the relevant standards set by the government. Green innovation achievements are highly uncertain and accompanied by high risk. Meanwhile, the inflow of innovation personnel will also bring additional adjustment costs to enterprises [33]. The implementation of command-control environmental regulation will increase the production cost of enterprises directly, aggravating the financing constraints faced by enterprises, and thus squeezing out and diverting the original innovation funds. In addition, based on the expensive regulation hypothesis, tighter regulation will accelerate bankruptcy of enterprises [34].

#### The impact of market-incentive environmental regulation on export technological sophistication

Through market-incentive environmental regulation, the government attempts to use economic leverage to adjust, guide, encourage, or support the economic activities of enterprises in accordance with the principles of economic cost-benefit. The essence is a transformation of the externality of the environment into an internality for enterprises, achieving the purpose of environmental goods privatization, through subsidies for a particular industry, pollution discharge trading policy, or other methods.

Instead of setting a pollutant discharge limit standard, the market-incentive environmental regulation uses the discharge fee or discharge right transaction as the main means. Assuming that a region collects environmental tax and the tax rate is *r*, the profit functions for Enterprises 1 and 2 can be modified as follows:

$$\pi_{1}=[m-n(q_{1}+q_{2})-z]q_{1}-(bq_{1}-s)r-as^{2}/2-A^{2}/2$$ (5)

$$\pi_{2}=[m-n(q_{1}+q_{2})-z]q_{2}-bq_{1}r$$ (6)

Where the cost for Enterprise 1 increases by (*bq*_1_*-s*)*r*, while the cost for Enterprise 2 increases by *bq*_1_*r*. Algebraic manipulation yields the analytic solution to the equilibrium output of the enterprise *q*_1_ = *q*_2_ = (*m-z-br*)/3*n*. The difference in profit between the two enterprises is then:

$$\Delta \pi_{m}=\pi_{1}-\pi_{2}=sr-as^{2}/2-A^{2}/2$$ (7)

Integrating *r = as* into Eq 7 transforms it into *Δπ*_*m*_ = π_*1*_*-π*_*2*_ = *as*^2^/2*-A*^2^/2, with the clear economic meaning that the difference in profit between Enterprise 1 and Enterprise 2 is equal to the extra profit and cost reduction due to the green innovation, minus the cost of the green innovation. Only when *Δπ*_*m*_>0 can the market-incentive environmental regulation promote green innovation and export competitiveness; the opposite will have an inhibitory effect on the latter.

On the one hand, market-incentive environmental regulation promotes manufacturing enterprises’ export technological sophistication through the mechanism of "innovation compensation" effect. Among the diverse means of regulation, the market-incentive environmental regulation can affect export technological sophistication through three channels. First, according to the signal transmission theory, governments convey a signal of marginal environmental cost by increasing enterprises’ pollution taxes. Enterprises will choose between green innovation to save marginal abatement costs and accepting the original high marginal abatement costs. When the increase in additional costs due to the environmental taxes leads to a decline in enterprises’ output and export competitiveness, enterprises will increase their green innovation efforts to promote export technological sophistication to ensure a stable market share. Second, market-incentive environmental regulation raises the price of some exports with pollution-intensive characteristics through environmental taxes or subsidies, and thus shifts the import demand preference of export destination countries to cheaper substitutes. Third, market-incentive environmental regulation can realize a transfer of wealth funds between enterprises and governments through pollutant discharge taxes and fees. As green innovation is a public product with a long research and development cycle and high risks, the government collects funds in the form of pollutant charges, and then transfers the funds to the innovation units through the establishment of a green innovation fund account, thus forming top-down technological innovation.

On the other hand, market-incentive environmental regulation can also inhibit enterprises’ export technological sophistication through the mechanism of "catering" effect. In the process of obtaining the initial distribution of pollutant discharge right, enterprises are motivated by rent-seeking; the high rent-seeking cost and government-enterprise relations are bound to have a negative impact on enterprises’ green activities [35]. In addition, for tax deduction, related industries will target this kind of industry for investment, with consequent excessive investment and production in competition for the support of the government for a particular industry and subsidy policy.

#### The impact of voluntary-participation environmental regulation on export technological sophistication

Voluntary-participation environmental regulation is the regulatory tool with the highest degree of freedom for enterprises; it is initiated by government, industrial organizations, or independent third parties to encourage and call enterprises in related industries to promise to improve the environment, health, and ecological safety.

In the case of voluntary-participation environmental regulation, the government and the public recognize enterprises with environmental awareness and environmental responsibility; this generates potential for hidden benefits, including corporate reputation and benign relations between governments and enterprises. Therefore, the basic model for voluntary-participation environmental regulation is modified as follows:

$$\pi_{1}=[m-n(q_{1}+q_{2})-z]q_{1}-as^{2}/2-A^{2}/2+I$$ (8)

$$\pi_{2}=[m-n(q_{1}+q_{2})-z]q_{2}-vI$$ (9)

$$s.t.\;bq_{1}-s=\bar{E}\;b\in [0,1]$$ (10)

where *I* denotes that enterprises accept the voluntary-participation environmental regulation to actively carry out green innovation, reduce pollution discharges, and convey to society a signal of enterprises with environmental responsibility, to obtain hidden benefits. *v* denotes the enthusiasm of the public for participation. Through algebraic calculation, the analytic solution to the equilibrium output corresponding to the two enterprises is obtained, *q*_1_ = (*m*-2*asb-z*)/3*n* and *q*_2_ = (*m+asb-z*)/3*n*, and the profit difference between the enterprises is

$$\Delta \pi_{p}=I+vX-(m+asb-z)asb/3n-as^{2}/2-A^{2}/2$$ (11)

According to Eq 11, when the public’s enthusiasm for environmental protection participation *v* increases, or when the enterprise’s hidden income generated by the regulation *I* increases, the profit difference between the enterprises *Δπ*_*p*_ will be higher. However, only when *Δπ*_*p*_>0 can voluntary-participation environmental regulation promote enterprises’ green innovation level and export competitiveness; otherwise, it may become a "greenwashing" tool for some enterprises in poor operation.

On the one hand, voluntary-participation environmental regulation can promote the export technological sophistication of manufacturing enterprises through the mechanism of "responsibility awareness" effect. First, according to the institution theory, some export enterprises tend to express "environment-friendly enterprises" signals to foreign consumers and investors, especially when export destination countries of consumers, producers, and other stakeholders, or multinational companies’ stakeholders are sensitive to the environmental behavior of enterprises [36, 37]. Second, the stronger an enterprise’s organizational resource acquisition ability and its existing environmental management and pollution prevention ability are, the stronger its motivation to use voluntary-participation environmental regulation will be [38]. Enterprises expect to generate invisible income through green innovation to achieve their economic interests or behavior, in line with moral standards. The establishment and improvement of public awareness of environmental protection will increase the "marginal willingness to pay" for scarce goods, e.g., environmental quality, and tolerance for goods produced by enterprises with high pollution and energy consumption will be greatly reduced, with consequent resistance behaviors [39, 40].

On the other hand, voluntary-participation environmental regulation also inhibits the export technological sophistication of manufacturing enterprises through the mechanism of "greenwashing" effect. There is a strong externality in the voluntary-participation environmental regulation, which requires an initial investment by enterprises, and the good reputation due to subsequent gains is shared by all enterprises involved. Thus, enterprises involved in voluntary-participation environmental regulation have a strong motivation to join but not to implement regulation requirements; their "free rider" behavior is also known as "greenwashing". [41] pointed out that social responsibility behavior was a burden to enterprises; environmental regulation, as an important part of corporate social responsibility, provides motivation for the generation of "greenwashing" behavior.

## Research design

### Econometric model specification

To investigate the impact of environmental regulation on the export technological sophistication of manufacturing enterprises, we develop the following benchmark econometric model:

$$\begin{array}{c} DTSI_{it}=\alpha_{1}+\beta_{0}ER_{c/p,t}+\beta_{1}ERS_{c/p,t}+\beta_{2}AGE_{it}+\beta_{3}SALE_{it}+\beta_{4}FS_{it}+\beta_{5}TFP_{it}\\ +\beta_{6}HHI_{it}+\beta_{7}SOE_{it}+\beta_{8}SUB_{it}+\beta_{9}MARKET_{pt}+\eta_{t}+\gamma_{i}+\varepsilon_{it} \end{array}$$ (12)

where the independent variables *ER*_*c/p*,*t*_ and *ERS*_*c/p*,*t*_ denote environmental regulation and its square term, respectively. The above model is an econometric regression model for the impact of command-control *OER*_*c*,*t*_, market-incentive *MER*_*p*,*t*_, and voluntary-participation *PER*_*p*,*t*_ environmental regulation on the export technological sophistication of manufacturing enterprises *DTSI*_*it*_. The dependent variable *DTSI*_*it*_ represents the export competitiveness of enterprises. The subscripts *i*, *c*, *p*, and *t* denote the enterprise, city, province, and year, respectively. To investigate the nonlinear effect of environmental regulation on export technological sophistication, the square terms corresponding to the various environmental regulations *OERS*_*ct*_, *MERS*_*pt*_, and *PERS*_*pt*_ are included. Control variables are specified as follows. *AGE*_*it*_ denotes the age of the enterprise, measured by the difference between each year and the founding year. *SALE*_*it*_ denotes the sales revenue of the enterprise, which is measured by the annual total sales of industrial products. *FS*_*it*_ denotes the size of the enterprise, which is measured by the total assets. *TFP*_*it*_ denotes the productivity of the enterprise, which is calculated by the LP method proposed by [42]. *HHI*_*it*_ denotes the industry concentration degree faced by the enterprise, which is measured by the Herfindahl index formula *HH*_*it*_ = ∑_i∈*Indj*_(*SALE*_*ist*_*/SALE*_*it*_)^2^ = ∑_i∈*Indj*_*MS*_*ist*_^2^. *SOE*_*it*_ denotes the ownership of state-owned enterprises, which is measured by a dummy variable: for state ownership, it is set to 1, otherwise, it set to 0. *SUB*_*it*_ denotes the subsidies received by the enterprise, which are measured by the total amount of subsidy received by the enterprise in the current year. *MARKET*_*pt*_ denotes the marketization level of the province, which is measured by the marketization index provided by [43], derived from a report "The Relative Process of Marketization Index in Different Regions of China in 2013". *η*_*t*_, *γ*_*i*_ and *ε*_*it*_ denote time fixed effects, enterprise fixed effects, and the error term, respectively.

The panel fixed effects regression with multi-dimensions is a method that is mainly used in the benchmark, heterogeneity, and mediating effect analysis section. The panel 2SLS (Two-Stage Least Squares) method and the Heckman two-step method [44] are used in the endogeneity analysis section. In addition, to alleviate the influence of heteroscedasticity, the related variables, except the dummy variable, were transformed into logarithm form. All empirical analysis results in this article are obtained through statistical software Stata 16.

#### Environmental regulation

In China, there are no relevant classified institutional issues, nor are there comprehensive environmental regulation indicators. In the actual environmental regulations, there is neither a fixed government intervention mode nor an independent regulation tool, which poses great difficulties for real measurement [45]. Based on the existing literature, we use a weighted linear summation method with high domain recognition to measure the intensity of heterogeneous environmental regulation.

With respect to command-control environmental regulation, five indicators were selected: "three simultaneous" project investment (It is the earliest environmental management system promulgated by China. According to regulation, pollution prevention facilities in construction projects should be designed, constructed, and put into operation at the same time as a main project is in progress), an industrial solid waste comprehensive utilization rate, a compliance rate for industrial wastewater discharge, a compliance rate for industrial sulfur dioxide emission, and a compliance rate for industrial smoke emission. For market-incentive environmental regulation, following [46], three indicators were selected: pollutant charge, annual investment of industrial pollution governance projects, and investment of pollution governance. For voluntary-participation environmental regulation, considering the availability of data, two single indicators were selected: the number of enterprises with environmental label product certification in each province and the number of environmental pollution and damage accident reports.

Following [47], the comprehensive index measure method for environmental regulation at the industrial level is extended to the regional level. Specifically, this study uses data on 384 cities, adopts the weighted linear sum method, and calculates the comprehensive index for command-control, market-incentive, and voluntary-participation environmental regulation in turn, based on the second-level single index of various environmental regulations. Three steps are involved:

The first step is to nondimensionalize the above single indicators successively:

$$ER_{irt}^{s}=\frac{ER_{irt}-MIN(ER_{it})}{MAX(ER_{it})-MIN(ER_{it})}$$ (13)

where the subscripts *r*, *i*, and *t* denote the region, enterprise, and year, respectively. *ER*_*irt*_ denotes the second-level single index of environmental regulation, *MAX*(•) and *MIN*(•) denote the maximum and minimum values of a single index at the regional level, respectively, and *ER*^*s*^_*irt*_ denotes the single index after nondimensionalization.

In the second step, we follow [48]: based on five single indicators at the regional level, an adjustment coefficient *M*_*irt*_ is calculated for each indicator. Considering that the degree of pollutant discharge in each region is different, the proportion of the degree of emission for each index is also different in the same area. Therefore, it is necessary to give different weights to each single index in different regions and years, to accurately reflect the change in regional pollution emissions intensity [49]. The adjustment coefficient is as follows,

$$M_{irt}=\frac{EMI_{irt}/\sum_{r}EMI_{irt}}{GDP_{rt}/\sum_{r}GDP_{rt}}$$ (14)

where the subscripts *i*, *r*, and *t* denote the individual, region, and year, respectively. *M*_*irt*_ denotes the ratio of the proportion of individual indicators’ discharge in the national total (*EMI*_*irt*_ /∑_*r*_*EMI*_*irt*_) to the proportion of regional GDP in the national total (*GDP*_*rt*_ /∑_*r*_*GDP*_*rt*_).

Third, based on the nondimensionalization of each single indicator *M*_*irt*_, the weighted average treatment of each single indicator obtains the comprehensive index of environmental regulation intensity for each district in each successive year.

$$ER_{rt}=\frac{1}{N}\sum_{i}^{N}M_{irt}\cdot ER_{irt}^{s}$$ (15)

#### Export technological sophistication

We expand and improve on [15]. First, the calculation of the export technological sophistication index in the existing literature is based on data at the national or industry level. The difference with our approach is that we attempt to measure export technological sophistication at the micro-enterprise level; we use the ratio of the value-added of enterprises in various industries to the total value-added as a weight. Second, the exports contain a considerable proportion of returned value-added and value-added abroad. In the calculation in the second step, if the weight is calculated directly, the export technological sophistication of enterprises is significantly biased. We use the proportion of export domestic value-added as a weight to measure the export technological sophistication of enterprises. Third, by considering robustness, we further revise the export technological sophistication measure.

In the first step, we calculate the export technological sophistication index at the product level:

$$PRODY_{pt}=\sum_{j}\left[(E_{pjt}/E_{jt})/(E_{pt}/E_{wt})\right]\cdot Y_{jt}$$ (16)

Where *p* denotes a HS code 6-digit product, *j* denotes country, *E*_*pj*_ denotes the exports of country *j*’s product *p*, *E*_*j*_ denotes the total exports of country *j*, *E*_*p*_ denotes the total world exports of products *p*, *E*_*w*_ denotes the total world exports, and *Y*_*j*_ denotes the gross domestic product per capita of country *j*. The weight is also known as the revealed comparative advantage index ∑_*j*_(*E*_*pj*_*/E*_*j*_)/(*E*_*pj*_/*E*_*w*_). For robustness, we follow [16] to adjust for the quality of the export technological sophistication. First, the relative price index at product level is adopted to measure the product quality, *Q*_*cpt*_ = *price*_*cpt*_/∑(*A*_*np*_•*price*_*npt*_), where *price*_*cpt*_ is the annual price of country *c*’s products *p* in year *t*, and *A*_*np*_ is the proportion of the exports of country *n*’s products *p* in the total exports of world’s product *p*. After the adjustment for quality, the export technological sophistication index at the product level is PRODY* = (*Q*_*cpt*_)^*λ*^•*PRODY*_*pt*_. Following [50], *λ* is set to 0.2.

In the second step, we calculate the export technological sophistication at the enterprise level:

$$DTSI0_{it}^{}=\sum_{p}(DVA_{ipt}/DVA_{it})\cdot PRODY_{pt}$$ (17)

$$DTSI1_{it}^{}=\sum_{p}(DVA_{ipt}/DVA_{it})\cdot PRODY_{pt}^{*}$$ (18)

$$ETSI1_{it}^{}=\sum_{p}(E_{ipt}/E_{it})\cdot PRODY_{pt}^{*}$$ (19)

$$ETSI0_{it}^{}=\sum_{p}(E_{ipt}/E_{it})\cdot PRODY_{pt}$$ (20)

Where *DTSI*0_*it*_ denotes enterprises’ export technological sophistication based on DVA (domestic value-added) without adjustment for quality, *DTSI*1_*it*_ denotes enterprises’ export technological sophistication based on DVA with adjustment for quality, *DVA*_*ip*_ denotes the export domestic value-added of enterprise *i*’s product *p*, *DVA*_*i*_ denotes the total domestic value-added from exports of enterprise *i*, and *DVA*_*ipt*_/*DVA*_*it*_ denotes the proportion of one kind of product’s DVA to all kinds of products’ DVA, which is used to measure the proportion of DVA export of products. For the calculation of the domestic value-added of enterprises’ exports, we follow [51] and [52]; we consider the issue of trade agents, capital goods import, indirect import of intermediate goods, and so on, and recalculate the index of domestic added value of enterprises’ exports (Due to space limitations, the steps for the relevant indicator construction and measurement are not included. For details, please refer to [51] and [52]). *ETSI*1_*it*_ denotes enterprises’ export technological sophistication based on enterprises’ exports with adjustment for quality, and *ETSI*0_*it*_ denotes enterprises’ export technological sophistication based on enterprises’ exports without adjustment for quality.

### Characteristic facts

#### Environmental regulation

Based on the regional standards, we highlight the differences in environmental regulation intensity, as shown in Table 1. Considering the respective mean values in the central-western and eastern regions, the command-control environmental regulation intensities are 0.759 and 0.639, the market-incentive environmental regulation intensities are 1.300 and 1.051, and voluntary-participation environmental regulation intensities are 2.152 and 1.565. Clearly, the environmental regulatory intensity, whether command-control, market-incentive, or voluntary-participation, is higher in the central-western region than in the eastern region. This may be related to the relatively backward production mode of the enterprises and the more serious environmental pollution problems in the central-western region.

**Table 1:** Regional differences in environmental regulation.

Year	Command-Control		Market-Incentive		Voluntary-Participation	
	Central-West	East	Central-West	East	Central-West	East
2004	1.117	0.878	1.082	0.780	2.683	1.068
2005	0.622	0.493	0.727	0.709	3.061	0.899
2006	0.658	0.512	2.299	1.139	3.347	1.404
2007	0.639	0.505	1.300	1.275	2.035	1.466
2008	1.063	0.927	1.260	1.453	1.353	1.861
2009	0.606	0.568	1.319	1.095	1.394	2.013
2010	0.608	0.589	1.114	0.903	1.193	2.246
Mean	0.759	0.639	1.300	1.051	2.152	1.565

#### Export technological sophistication

We present preliminary annual differences between four export technological sophistication indicators, based on enterprise trade pattern, location, and ownership, in Table 2. We compare the export technological sophistication of various enterprises. First, taking the export DVA value as an example, the average export technological sophistication for ordinary trade enterprises and processing trade enterprises is 3.957 and 3.816, respectively, which shows that the sophistication level of ordinary trade enterprises is higher than that of processing trade enterprises. For central-western enterprises, it is higher than that of processing trade enterprises. The average export technological sophistication levels for domestic enterprises and eastern enterprises are 3.915 and 3.867, respectively, which indicates that the sophistication level of eastern enterprises is higher than that of central-western enterprises. The average export technological sophistication levels of domestic enterprises and foreign-funded enterprises are 3.904 and 3.849, respectively, indicating that the sophistication level of domestic enterprises is higher than that of foreign enterprises. Third, taking the export technological sophistication index with exports-weight and quality-adjustment as an example, the average indexes for ordinary trade enterprises and processing trade enterprises are 4.081 and 3.951, respectively, which indicates that the sophistication level of the ordinary trade enterprises is higher than that of the processing trade enterprises. The average indexes for domestic and foreign-funded enterprises are 4.025 and 4.001, respectively, which indicates that the sophistication level of the central-western is higher than that of the eastern enterprises. The average indexes for domestic and foreign-funded enterprises are 4.034 and 3.979, respectively, indicating that the sophistication level of domestic is higher than that of foreign-funded enterprises. The conclusion is consistent with the standards of enterprise revised DVA and enterprise revised export technological sophistication.

**Table 2:** Export technological sophistication during 2004–2010.

Type	Ordinary				Processing			
Year	*TSI0*	*TSI1*	*TSI8*	*TSI9*	*TSI0*	*TSI1*	*TSI8*	*TSI9*
2004	4.064	3.933	3.993	3.869	3.927	3.754	3.815	3.643
2005	4.067	3.876	3.996	3.807	3.949	3.735	3.834	3.623
2006	4.097	3.958	4.025	3.887	3.958	3.821	3.858	3.721
2007	4.139	4.007	4.067	3.935	3.981	3.858	3.869	3.747
2008	4.094	3.966	4.019	3.892	3.967	3.815	3.855	3.706
2009	4.085	3.991	4.018	3.927	3.974	3.886	3.846	3.740
2010	4.013	3.956	3.930	3.875	3.893	3.822	3.771	3.701
Mean	4.081	3.957	4.008	3.886	3.951	3.816	3.836	3.700
Type	Central-West				East			
Year	TSI0	TSI1	TSI8	TSI9	TSI0	TSI1	TSI8	TSI9
2004	3.988	3.877	3.885	3.789	3.976	3.828	3.801	3.662
2005	3.999	3.824	3.910	3.737	3.989	3.788	3.818	3.620
2006	4.030	3.896	3.932	3.801	4.013	3.872	3.855	3.716
2007	4.071	3.962	3.987	3.858	4.056	3.925	3.903	3.773
2008	4.048	3.928	3.953	3.838	4.015	3.879	3.872	3.741
2009	4.037	3.953	3.959	3.880	4.009	3.881	3.874	3.779
2010	3.994	3.947	3.904	3.859	3.944	3.883	3.789	3.730
Mean	4.025	3.915	3.934	3.826	4.001	3.867	3.846	3.721
Type	Domestic				Foreign			
Year	TSI0	TSI1	TSI8	TSI9	TSI0	TSI1	TSI8	TSI9
2004	4.019	3.895	3.918	3.802	3.953	3.798	3.743	3.596
2005	4.026	3.836	3.924	3.738	3.968	3.765	3.764	3.564
2006	4.054	3.915	3.958	3.822	3.987	3.847	3.793	3.652
2007	4.088	3.960	3.994	3.862	4.030	3.901	3.829	3.701
2008	4.045	3.916	3.943	3.816	3.988	3.848	3.803	3.672
2009	4.027	3.878	3.949	3.858	3.995	3.901	3.804	3.710
2010	3.973	3.916	3.867	3.812	3.926	3.865	3.729	3.672
Mean	4.034	3.904	3.938	3.817	3.979	3.849	3.782	3.655

**Fig 2:**
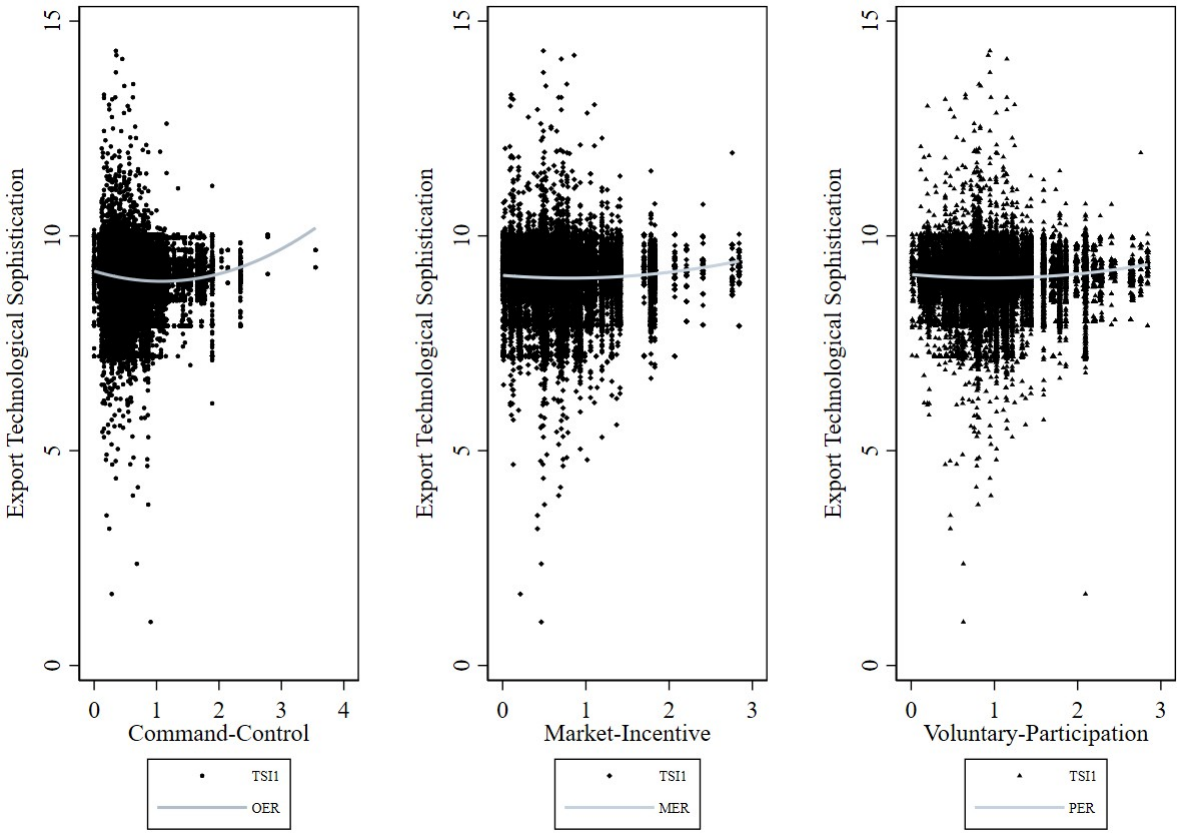
Scatter diagram of environmental regulation and export technological sophistication during 2004–2010.

A scatter diagram reflecting the relationship between environmental regulation and export technological sophistication for manufacturing enterprises is presented in Fig 2. Clearly, command-control, market-incentive, and voluntary-participation environmental regulations and export technological sophistication exhibit a U-shaped nonlinear relationship: environmental regulation intensity initially (beginning of the ascension) inhibits export technological sophistication; however, when environmental regulation intensity reaches a certain threshold, the regulation improves export technological sophistication. However, it is worth noting that, when control variables and fixed effects are ignored, the reference value of the variable relationship presented is limited; therefore more complex and comprehensive methods are necessary for an in-depth investigation of the variable relationship.

### Data sources and processing

The research sample period is from 2004 to 2010, and six sets of data were used. The first dataset came from the Chinese Industrial Enterprise Database. To deal with problems such as sample mismatch, missing indicators, and abnormal indicators, we follow [53]: (1) We eliminate enterprises with fewer than eight employees, (2) we exclude enterprises with negative total assets, total fixed assets, intermediate inputs and payable wages, and (3) we exclude enterprises founded before 1949. The second dataset came from the China Customs Trade Database. Due to the problem with Chinese enterprises’ reliance on trade agents in the import and export business, [54]’s method was used: the enterprise name contained in the "Import and Export", "Commercial Trade", "Technological Trade", "Industrial Trade", "Trade" and "Foreign Commerce" words of observations was used, and the monthly data aggregation to annual data eliminates abnormal observations. The third dataset came from the World Input-Output Database. According to the input and output information between the states and the sectors, the KWW model [55] was used to decompose and calculate the return value-added rate for each industry and the value-added rate for export. The fourth dataset was obtained from the China Urban Statistical Yearbook and China Environmental Statistical Yearbook, from which a single indicator is selected to construct environmental regulation. The fifth dataset came from the Chinese Enterprise Patent Database. The green invention innovation, green utility innovation, and green design innovation variables were identified. The sixth dataset came from the UN-Comtrade Database. In this study, 5224 products from 141 countries were selected to calculate the export technological sophistication of HS code 6-digit level products. Among the command-control variables, enterprise age is expressed as the difference between each year and the year of establishment of the enterprise, while the other variables are obtained directly from the Chinese Industrial Enterprise Database. The marketization index is from the China National Economic Research Institute.

Since the data are distributed across multiple databases, a matching merge is required before use. Consistent with [56] and [57], the Industrial Enterprise Database is merged with enterprise code and enterprise name in different years respectively, and then matched and merged with the Customs Trade Database based on the enterprise name. Finally, the data were merged with other data needed for combination. Merging with the World Input-Output Database was based on the industry and year variable; merging with the urban statistical yearbook and statistical yearbook environmental data was based on the provinces, cities, and year variables; merging with the China Patent Database data was based on the enterprise name and year variables; merging with the UN-Comtrade Database data was based on the year and 6-digits HS code; merging with the marketization index data was based on the province and year variables. In terms of coding processing, the HS2017 standard was used to unify the data related to customs HS codes through the code conversion table provided by the UN Statistics Division. The BEC-Rev. 4 standard was used for the classification of final products, capital goods, and intermediate products, while the SITC-Rev. 3 standard was used for the classification of industry types.

**Fig 3:**
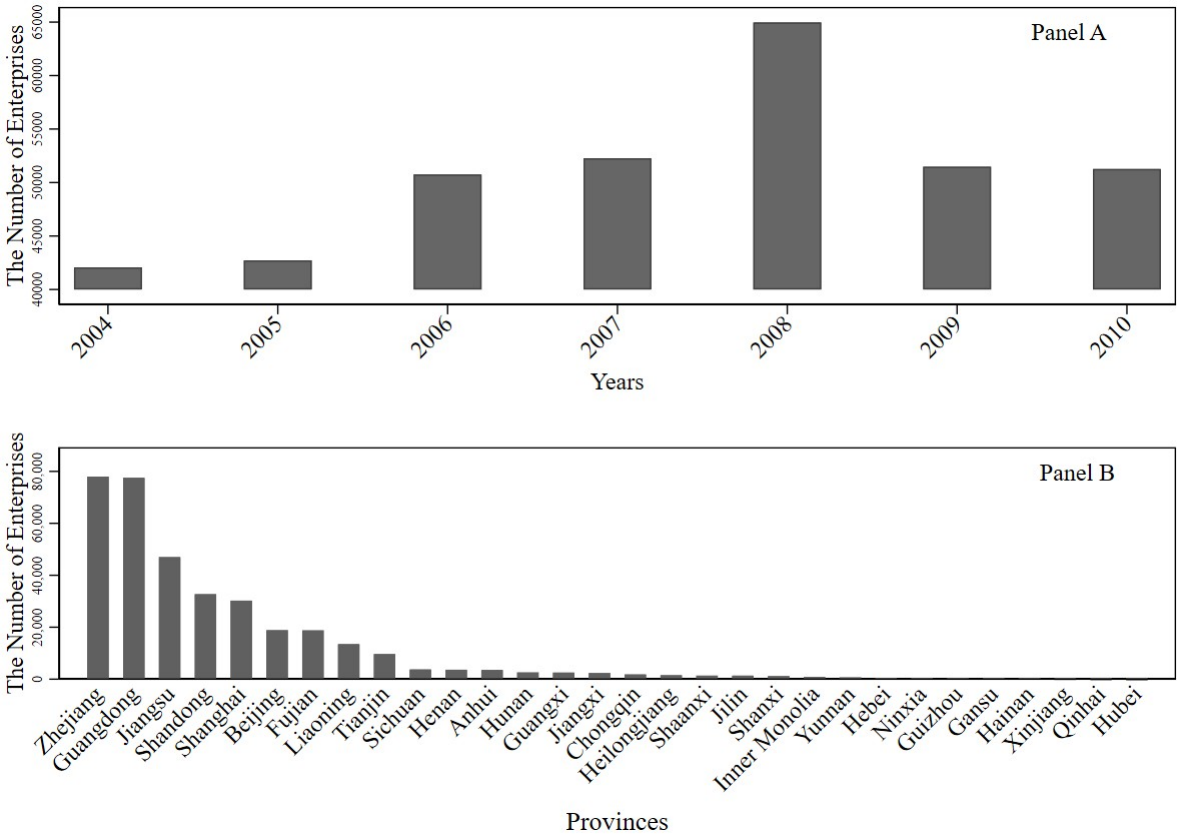
Distribution of manufacturing enterprises across years and provinces.

We report the distribution of manufacturing enterprises in the consolidated data shown in Fig 3. Panel A reports the distribution of manufacturing enterprises in each year, while Panel B reports the distribution of manufacturing enterprises in China’s provinces (municipalities or autonomous regions).

The descriptive statistics are presented in Table 3.

**Table 3:** Descriptive statistics.

Variable	Obs.	Mean	S.D.	Median	Min	Max
*DTSI0*	354313	7.563	3.684	9.449	0	14.33
*DTSI1*	354263	7.33	3.569	9.034	0	14.31
*OER*	354849	0.465	0.268	0.404	0	3.549
*MER*	354849	0.663	0.382	0.649	0.004	2.841
*PER*	354849	0.892	0.391	0.817	0.011	2.841
*AGE*	354849	3.246	0.053	3.262	3.098	3.293
*MARKET*	354849	2.218	0.148	2.219	1.261	2.478
*HHI*	354849	0.415	0.228	0.405	0.004	0.693
*SALE*	354849	10.66	1.386	10.51	0.693	19.07
*FS*	354849	10.38	1.501	10.23	3.434	20.16
*TFP*	354849	0.305	0.133	0.342	0	0.999
*SOE*	354849	0.036	0.187	0	0	1
*SUB*	354849	0.001	0.013	0	0	2.897
*RD1*	354849	0.003	0.068	0	0	4.635
*RD2*	354849	0.077	0.363	0	0	6.288
*RD3*	354849	0.037	0.286	0	0	6.82
*AURCUR*	354849	2.172	0.042	2.177	1.665	2.298

## Empirical results and analysis

### Benchmark results analysis

The benchmark results pertaining to the analysis of the impact of environmental regulation on the export technological sophistication of enterprises are shown in Table 4. The dependent variables are export technological sophistication with the traditional measure and export technological sophistication with the adjusted measure.

**Table 4:** Benchmark regression results.

variable	(1)	(2)	(3)	(4)	(5)	(6)
	*DTSI0*	*DTSI1*	*DTSI0*	*DTSI1*	*DTSI0*	*DTSI1*
*OER*	-0.601***	-0.722***				
	(0.23)	(0.24)				
*OERS*	0.191*	0.230**				
	(0.10)	(0.10)				
*MER*			0.740***	0.865***		
			(0.23)	(0.26)		
*MERS*			-0.247***	-0.284***		
			(0.09)	(0.10)		
*PER*					0.730***	0.863***
					(0.17)	(0.20)
*PERS*					-0.226***	-0.260***
					(0.06)	(0.08)
*AGE*	-0.315***	-0.286***	-0.319***	-0.291***	-0.326***	-0.299***
	(0.02)	(0.02)	(0.02)	(0.02)	(0.02)	(0.02)
*MARKET*	-0.354*	-0.610***	-0.221	-0.447***	-0.033	-0.222
	(0.20)	(0.18)	(0.15)	(0.13)	(0.14)	(0.13)
*TFP*	-1.509***	-1.435***	-1.515***	-1.442***	-1.522***	-1.451***
	(0.17)	(0.16)	(0.17)	(0.16)	(0.17)	(0.16)
*HHI*	0.258***	0.243***	0.254***	0.238***	0.263***	0.249***
	(0.07)	(0.07)	(0.07)	(0.07)	(0.07)	(0.07)
*SALE*	0.010	-0.002	-0.000	-0.015	0.008	-0.005
	(0.03)	(0.03)	(0.03)	(0.03)	(0.03)	(0.03)
*FS*	-0.226***	-0.283***	-0.335***	-0.415***	-0.210***	-0.263***
	(0.07)	(0.07)	(0.08)	(0.09)	(0.06)	(0.06)
*SOE*	-0.310**	-0.354***	-0.302**	-0.345**	-0.284**	-0.324**
	(0.14)	(0.13)	(0.15)	(0.14)	(0.14)	(0.13)
*SUB*	-0.145	-0.290	-0.188	-0.342	-0.148	-0.293
	(0.55)	(0.53)	(0.54)	(0.52)	(0.55)	(0.53)
Constant	14.550***	14.465***	13.975***	13.776***	13.427***	13.120***
	(1.06)	(1.06)	(1.16)	(1.17)	(1.07)	(1.07)
U-shaped test	1.54*	1.80**	2.33***	2.30**	2.81***	2.42***
P value	0.062	0.037	0.010	0.011	0.003	0.008
Reflection point	1.573	1.570	1.498	1.523	1.615	1.660
Year fixed effect	Yes	Yes	Yes	Yes	Yes	Yes
Enterprise fixed effect	Yes	Yes	Yes	Yes	Yes	Yes
F value	56.73	52.95	54.06	49.21	53.05	50.11
*R-squared*	0.682	0.680	0.682	0.680	0.682	0.681
Obs.	326079	326032	326079	326032	326079	326032

Note: The levels of ***, ** and * are significant at 1%, 5% and 10%, respectively. The model controls the year and enterprise fixed effect, the standard error presented in parenthesis adopted at enterprise level clustering robustness. The joint significance test F value shows that all variables of the model are overall significant. The results of goodness of fit *R-squared* show that the model explains about 68% of the dependent variable changes as a whole, which is in a reasonable range.

Columns (1)-(2) present the results relating to the impact of command-control environmental regulation on export technological sophistication. The coefficients of *OER* and *OERS* are -0.601 and 0.191 with the traditional measure, and -0.722 and 0.230 with the adjusted measure, respectively. These are significant at the 5%-10% levels of significance. The U-shaped test values are 1.54 and 1.80 for the traditional and adjusted measures, respectively, both significant at the 5%-10% levels of significance, which is similar to the J-shaped curve characteristics of [58]. This is indicative of the U-shape nonlinearity of the impact of command-control environmental regulation on export technological sophistication. We follow [59] to examine the nonlinearity.

Columns (3)-(4) present the results relating to the impact of market-incentive environment regulation on export technological sophistication. The coefficients of *MER* and *MERS* are 0.740 and -0.247 for the traditional measure, 0.865 and -0.284 for the adjusted measure, respectively, and significant at the 1% level of significance. The U-shaped test values are 2.33 and 2.30 for the traditional and adjusted measures, respectively, and significant at the 1%-5% levels of significance, suggesting that the impact of market-incentive environmental regulation on export technological sophistication assumes inverted U-shaped nonlinear characteristics.

Columns (5)-(6) present the results relating to the impact of voluntary-participation environmental regulation on export technological sophistication. The coefficients of *PER* and *PERS* are 0.730 and -0.226 for the traditional measure, and 0.863 and -0.260 for the adjusted measure, respectively, and significant at the 1% level of significance. The U-shaped test values are 2.81 and 2.42 for the traditional and adjusted measures, respectively, and significant at the 1% level of significance, showing that the impact of voluntary-participation environmental regulation on export technological sophistication exhibits inverted U-shaped nonlinear characteristics.

Further analysis shows that the inflection points of the impact of command-control environmental regulation on export technological sophistication are 1.573 and 1.570 for the traditional and adjusted measures, respectively, while the overall level of China’s command-control environmental regulation intensity is 0.465; this intensity level has not gone beyond the inflection point of the nonlinear effect, and is still in the negative impact interval. The inflection points for the impact of market-incentive environmental regulation are 1.498 and 1.523 for the traditional and adjusted measures, respectively, while the overall level of China’s market-incentive environmental regulation intensity is 0.663, which has not extended beyond the inflection point of the nonlinear impact and is still in the positive impact interval. The inflection points for the impact of voluntary-participation environmental regulation are 1.615 and 1.660 for the traditional and adjusted measures, respectively, while the overall level of China’s voluntary-participation environmental regulation intensity is 0.891, which has not extended beyond the inflection point of the nonlinear impact and is still in the positive interval.

### Heterogeneity results

The sample analysis is conducted from three perspectives: trade pattern, region, and ownership.

#### The heterogeneity of enterprises’ trade pattern

Following [52], we divided the sample into ordinary trade, processing trade, and mixed trade enterprises. In Table 5, the results showed that the impact of environmental regulation on the export technological sophistication of enterprises with different trade patterns presented U-shaped or inverted U-shaped nonlinearities, e.g., the U-shaped characteristics of command-control environmental regulation and export competitiveness of mixed trade enterprises.

**Table 5:** Regression results of the heterogeneity in trade patterns.

Variable	(1)	(2)	(3)	(4)	(5)	(6)	(7)	(8)	(9)
	Ordinary	Processing	Mixed	Ordinary	Processing	Mixed	Ordinary	Processing	Mixed
*OER*	0.010	-0.854	-0.738***						
	(0.18)	(0.68)	(0.22)						
*OERS*	-0.019	0.413	0.275***						
	(0.08)	(0.43)	(0.10)						
*MER*				0.352***	1.799***	0.847**			
				(0.11)	(0.40)	(0.38)			
*MERS*				-0.091*	-0.714***	-0.309**			
				(0.05)	(0.19)	(0.14)			
*PER*							0.409***	1.100**	0.743***
							(0.11)	(0.56)	(0.26)
*PERS*							-0.127***	-0.404*	-0.242**
							(0.04)	(0.23)	(0.10)
Constant	10.900***	10.990***	10.682***	13.259***	13.244***	12.024***	12.491***	11.796***	11.274***
	(1.01)	(1.16)	(1.11)	(1.39)	(1.09)	(1.08)	(1.58)	(1.69)	(1.59)
Control var.	Yes	Yes	Yes	Yes	Yes	Yes	Yes	Yes	Yes
U-shaped test	0.05	0.92	2.40***	0.86	3.03**	1.57*	2.47***	2.07**	2.15**
P value	0.478	0.18	0.009	0.194	0.001	0.059	0.007	0.020	0.017
Reflection point			1.342		1.260	1.371	1.610	1.361	1.535
Year fixed effect	Yes	Yes	Yes	Yes	Yes	Yes	Yes	Yes	Yes
Enterprise fixed effect	Yes	Yes	Yes	Yes	Yes	Yes	Yes	Yes	Yes
F value	24.68	25.57	28.14	11.77	33.78	24.92	30.62	31.97	25.54
*R-squared*	0.631	0.632	0.632	0.813	0.813	0.813	0.792	0.792	0.792
Obs.	173464	31579	78063	173464	31579	78063	173464	31579	78063

Note: The levels of ***, ** and * are significant at 1%, 5% and 10%, respectively. The model controls the year and enterprise fixed effect, the standard error presented in parenthesis adopted at enterprise level clustering robustness. The joint significance test F value shows that all variables of the model are overall significant. The result of goodness of fit *R-squared* shows that the model as a whole explains at least 63% of the dependent variable changes, which is in a reasonable interval.

Furthermore, the heterogeneity is investigated by considering inflection points. Comparing the inflection point with the mean value of environmental regulation for each set of results, we find a negative correlation between command-control environmental regulation and the export technological sophistication of mixed trade enterprises. In addition, market-incentive and voluntary-participation environmental regulations had positive correlations with the export technological sophistication of enterprises with all types of trade patterns.

#### The heterogeneity of enterprises’ location

Based on the subsample standards of the National Bureau of Statistics, the samples were divided into enterprises in the central-western regions and eastern regions according to the regional divisions of enterprises. In detail, according to the National Bureau of Statistics, the eastern regions of China are Beijing, Tianjin, Hebei, Shanghai, Jiangsu, Zhejiang, Fujian, Shandong, Guangdong, and Hainan; other provinces, municipalities, and other autonomous regions were divided into middle-western regions (including three provinces of the northeastern region). The relevant heterogeneous regression results are shown in Table 6.

**Table 6:** Regression results of the heterogeneity in located region.

Variable	(1)	(2)	(3)	(4)	(5)	(6)
	Central-western	Eastern	Central-western	Eastern	Central-western	Eastern
*OER*	-0.493	-0.804***				
	(0.34)	(0.28)				
*OERS*	0.086	0.286**				
	(0.11)	(0.12)				
*MER*			0.375**	0.912**		
			(0.17)	(0.37)		
*MERS*			-0.184***	-0.252		
			(0.06)	(0.15)		
*PER*					0.162	0.790***
					(0.15)	(0.25)
*PERS*					-0.103**	-0.175*
					(0.05)	(0.10)
Constant	14.180***	13.580***	13.657***	14.486***	13.817***	13.002***
	(1.28)	(1.25)	(1.24)	(1.20)	(1.34)	(1.18)
Control var.	Yes	Yes	Yes	Yes	Yes	Yes
U-shaped test	0.23	2.15**	1.06	2.05***	0.98	0.66
P value	0.409	0.017	0.145	0.022	0.165	0.256
Reflection point		1.406				
Year fixed effect	Yes	Yes	Yes	Yes	Yes	Yes
Enterprise fixed effect	Yes	Yes	Yes	Yes	Yes	Yes
F value	14.81	15.94	14.57	50.90	41.22	38.45
*R-squared*	0.772	0.772	0.772	0.753	0.753	0.753
Obs.	37759	37759	37759	287754	287754	287754

Note: The levels of ***, ** and * are significant at 1%, 5% and 10%, respectively. The model controls the year and enterprise fixed effect, the standard error presented in parenthesis adopted at enterprise level clustering robustness. The joint significance test F value shows that all variables of the model are overall significant. The goodness of fit *R-squared* results show that the model as a whole explains at least 75% of the dependent variable changes, which is in a reasonable interval.

The results show that there was a heterogeneous nonlinearity for environmental regulation on the export technological sophistication of enterprises in different regions. For some results, however, there were monotone characteristics, e.g., market-incentive environmental regulation in the central-western and eastern regions. By reference to inflection points, we found that command-control environmental regulation negatively correlated with the export technological sophistication of enterprises in the eastern regions, while market-incentive and voluntary-participation environmental regulations positively correlated with the export technological sophistication of enterprises in each region to some extent.

#### The heterogeneity of ownership

The enterprises that belonged to China-foreign cooperative enterprises, China-foreign joint ventures, and wholly foreign-owned enterprises were classified as foreign-funded enterprises, while the other sample enterprises were classified as domestic-funded. The relevant empirical results are shown in Table 7.

**Table 7:** Empirical results of the heterogeneity in ownership.

Variable	(1)	(2)	(3)	(4)	(5)	(6)
	Domestic	Foreign	Domestic	Foreign	Domestic	Foreign
*OER*	-0.339*	-0.998***				
	(0.20)	(0.26)				
*OERS*	0.140	0.291**				
	(0.09)	(0.11)				
*MER*			0.416***	1.251***		
			(0.15)	(0.40)		
*MERS*			-0.154***	-0.409**		
			(0.06)	(0.16)		
*PER*					0.580***	0.995***
					(0.12)	(0.27)
*PERS*					-0.197***	-0.288***
					(0.05)	(0.10)
Constant	13.977***	13.701***	13.373***	14.904***	13.881***	12.994***
	(1.19)	(1.28)	(1.24)	(1.07)	(1.21)	(1.09)
Control var.	Yes	Yes	Yes	Yes	Yes	Yes
U-shaped test	1.47*	2.47***	3.02***	1.87**	2.04**	2.00**
P value	0.071	0.007	0.001	0.032	0.021	0.024
Reflection point		1.710	1.351	1.529	1.472	1.727
Year fixed effect	Yes	Yes	Yes	Yes	Yes	Yes
Enterprise fixed effect	Yes	Yes	Yes	Yes	Yes	Yes
F value	24.47	26.07	25.03	85.30	69.51	85.13
*R-squared*	0.755	0.755	0.755	0.748	0.748	0.748
Obs.	142289	142289	142289	183555	183555	183555

Note: The levels of ***, ** and * are significant at 1%, 5% and 10%, respectively. The model controls the year and enterprise fixed effect, the standard error presented in parenthesis adopted at enterprise level clustering robustness. The joint significance test F value shows that all variables of the model are overall significant. The goodness of fit *R-squared* results show that the model as a whole explains at least 74% of the dependent variable changes, which is in a reasonable interval.

The results show that there was a heterogeneous nonlinearity for environmental regulation on the export technological sophistication of enterprises with different ownership structures. Furthermore, by reference to inflection points, we found that command-control environmental regulation was negatively correlated with the export technological sophistication of foreign-funded enterprises, while market-incentive and voluntary-participation environmental regulations were positively correlated with the export technological sophistication of domestic-funded and foreign-funded enterprises.

### Mediating effect analysis

This section examines the mediating mechanism of heterogeneous environmental regulation on export technological sophistication. We follow [60], who proposed a stepwise regression method to test for the effect of mediation, with the coefficient of interaction as the variable of interest. However, in practice, while interaction coefficients may be significant, individual variables do not pass the significance test [61, 62]. Therefore, in the sequential test, either one independent variable or a mediating variable is insignificant; the [63] method should be used to further test the interaction coefficient to determine whether there is a mediating effect.

Based on the above explanation of the mediating effect test, the relevant econometric model paradigm is set as follows:

$$\begin{array}{ll} DTSI1_{it} & =\alpha_{0}+\beta_{0}ER_{rt}+\beta_{1}ERS_{rt}+\beta_{2}AGE_{it}+\beta_{3}SALE_{it}+\beta_{4}FS_{it}+\beta_{5}TFP_{it}\\ & +\beta_{6}HHI_{ct}+\beta_{7}SOE_{it}+\beta_{8}SUB_{it}+\beta_{9}MARKET_{pt}\\ & +\eta_{t}+\gamma_{i}+\varepsilon_{it} \end{array}$$ (21)

$$\begin{array}{ll} RD_{it} & =\alpha_{0}+\beta_{0}ER_{rt}+\beta_{1}ERS_{rt}+\beta_{2}AGE_{it}+\beta_{3}SALE_{it}+\beta_{4}FS_{it}+\beta_{5}TFP_{it}\\ & +\beta_{6}HHI_{ct}+\beta_{7}SOE_{it}+\beta_{8}SUB_{it}+\beta_{9}MARKET_{pt}\\ & +\eta_{t}+\gamma_{i}+\varepsilon_{it} \end{array}$$ (22)

$$\begin{array}{ll} DTSI_{it} & =\alpha_{0}+\beta_{0}ER_{rt}+\beta_{1}ERS_{rt}+\beta_{2}RD_{it}+\beta_{3}AGE_{it}+\beta_{4}SALE_{it}+\beta_{5}FS_{it}\\ & +\beta_{6}TFP_{it}+\beta_{7}HHI_{ct}+\beta_{8}SOE_{it}+\beta_{9}SUB_{it}+\beta_{10}MARKET_{pt}\\ & +\eta_{t}+\gamma_{i}+\varepsilon_{it} \end{array}$$ (23)

where *ER*_*rt*_ denotes environmental regulation intensity, which is composed of command-control, market-incentive, and voluntary-participation environmental regulation. *RD*_*it*_ denotes the enterprises’ green innovation, composed of green invention innovation, green utility innovation, and green design innovation. Indeed, the different types of green innovation represent three different levels of independent innovation capabilities and different competitive strategies. Green invention innovation represents the innovation type with the highest technology content, followed by green utility innovation, while green design innovation represents the lowest technical level [64]. The ratio of the number of green patent applications to the total number of patent applications at the city level is used as a weight and multiplied by the number of enterprise patent applications to estimate the level of green innovation at the enterprise level. The relevant data is from the China Statistical Yearbook.

Table 8 shows the mediating mechanism regression results for the impact of command-control environmental regulation. Column (1) shows the regression result of the total effect, while Columns (2)-(3), (4)-(5), and (6)-(7) show the results of the mediating effect of green invention, green utility, and green design innovation, respectively. The results in Columns (4) and (5) indicate that the green utility innovation of enterprises is indeed a mediating channel for the impact of command-control environmental regulation on export technological sophistication. The [63] method was introduced to conduct the mediating effect test: the absolute value of the z statistics revealed that green invention innovation was another mediation channel. Green invention innovation contains the highest level of technology, which represents the highest level of green innovation ability for enterprises; it also requires more green innovation investment from enterprises. Command-control environmental regulation has a significant negative effect on export technological sophistication, which is the most important factor to restrain the latter.

**Table 8:** The mediating effect of command-control environmental regulation.

Variable	(1)	(2)	(3)	(4)	(5)	(6)	(7)
	*DTSI1*	*RD1*	*DTSI1*	*RD2*	*DTSI1*	*RD3*	*DTSI1*
*OER*	-0.722***	-0.006***	-0.722***	-0.140***	-0.714***	-0.042***	-0.721***
	(0.08)	(0.00)	(0.08)	(0.01)	(0.08)	(0.01)	(0.08)
*OERS*	0.230***	0.002*	0.230***	0.033***	0.228***	0.015***	0.230***
	(0.05)	(0.00)	(0.05)	(0.01)	(0.05)	(0.01)	(0.05)
*RD1*			0.032				
			(0.07)				
*RD2*					0.060***		
					(0.01)		
*RD3*							0.019
							(0.02)
Constant	14.465***	0.024***	14.464***	0.470***	14.437***	0.054**	14.464***
	(0.20)	(0.01)	(0.20)	(0.03)	(0.20)	(0.02)	(0.20)
Control var.	Yes	Yes	Yes	Yes	Yes	Yes	Yes
Year fixed effect	Yes	Yes	Yes	Yes	Yes	Yes	Yes
Enterprise fixed effect	Yes	Yes	Yes	Yes	Yes	Yes	Yes
F value	669.3	7.494	608.5	288.8	610.2	14.33	608.6
*R-squared*	0.680	0.270	0.680	0.381	0.680	0.257	0.680
Obs.	326032	326620	326032	326620	326032	326620	326032

Note: The levels of ***, ** and * are significant at 1%, 5% and 10%, respectively. The model controls the year and enterprise fixed effect, the standard error presented in parenthesis adopted at enterprise level clustering robustness. The joint significance test F value shows that all variables of the model are overall significant. The goodness of fit *R-squared* results show that the models explain the changes of dependent variables to varying degrees, and they are all reasonable for the panel model.

Table 9 shows the mediating mechanism regression results of the impact of market-incentive environmental regulation. The regression models are similar to those in Table 8. The results in Columns (4) and (5) indicate that the green utility innovation of enterprises is indeed a mediation channel through which the market-incentive environmental regulation affects export technological sophistication. The absolute value of the z statistics in the [63] method reveals that green invention innovation and green design innovation are other mediating channels. The direction of the coefficients shows that the "innovation compensation" effect of market-incentive environmental regulation is stronger than the "pandering" effect; the "perceptive responsibility” effect of voluntary-participation environmental regulation is stronger than the "greenwashing" effect.

**Table 9:** The mediating effect of market-incentive environmental regulation.

Variable	(1)	(2)	(3)	(4)	(5)	(6)	(7)
	*DTSI1*	*RD1*	*DTSI1*	*RD2*	*DTSI1*	*RD3*	*DTSI1*
*MER*	0.8648***	0.0000	0.8648***	0.0563***	0.8613***	0.0067	0.8647***
	(0.060)	(0.002)	(0.060)	(0.009)	(0.060)	(0.007)	(0.060)
*MERS*	-0.2840***	-0.0003	-0.2840***	-0.0178***	-0.2829***	-0.0006	-0.2840***
	(0.028)	(0.001)	(0.028)	(0.004)	(0.028)	(0.003)	(0.028)
*RD1*			0.0397				
			(0.074)				
*RD2*					0.0623***		
					(0.014)		
*RD3*							0.0207
							(0.016)
Constant	13.7759***	0.0163***	13.7752***	0.2838***	13.7582***	0.0086	13.7757***
	(0.186)	(0.005)	(0.186)	(0.027)	(0.186)	(0.023)	(0.186)
Control var.	Yes	Yes	Yes	Yes	Yes	Yes	Yes
Year fixed effect	Yes	Yes	Yes	Yes	Yes	Yes	Yes
Enterprise fixed effect	Yes	Yes	Yes	Yes	Yes	Yes	Yes
F value	683.1	6.350	621.0	259.7	622.9	11.81	621.1
*R-squared*	0.680	0.270	0.680	0.380	0.681	0.256	0.680
Obs.	326032	326620	326032	326620	326032	326620	326032

Note: The levels of ***, ** and * are significant at 1%, 5% and 10%, respectively. The joint significance test F value shows that all variables of the model are overall significant. The goodness of fit *R-squared* results show that the models explain the changes of dependent variables to varying degrees, and they are all reasonable for the panel model.

Table 10 shows the mediating mechanism regression results of the impact of voluntary-participation environmental regulation. The results in Columns (4) and (5) indicate that green utility innovation is indeed a mediating channel through which voluntary-participation environmental regulation affects export technological sophistication. The absolute value of the z statistics in the [63] method reveals that green invention innovation is another mediating channel; the "perceptive responsibility” effect of voluntary-participation environmental regulation is stronger than the "greenwashing" effect.

**Table 10:** The mediating effect of voluntary-participation environmental regulation.

Variable	(1)	(2)	(3)	(5)	(6)	(8)	(9)
	*DTSI1*	*RD1*	*DTSI1*	*RD2*	*DTSI1*	*RD3*	*DTSI1*
*PER*	0.8626***	0.0049***	0.8625***	0.8576***	-0.0059	0.8626***	0.0049***
	(0.047)	(0.001)	(0.047)	(0.047)	(0.006)	(0.047)	(0.001)
*PERS*	-0.2601***	-0.0015***	-0.2600***	-0.2585***	0.0025	-0.2601***	-0.0015***
	(0.020)	(0.001)	(0.020)	(0.020)	(0.002)	(0.020)	(0.001)
*RD1*			0.0208				
			(0.074)		0.0552***		
*RD2*					(0.014)		
*RD3*							0.0222
							(0.016)
Constant	13.1200***	0.0143***	13.1197***	13.1077***	0.0070	13.1200***	0.0143***
	(0.187)	(0.005)	(0.187)	(0.187)	(0.023)	(0.187)	(0.005)
Control var.	Yes	Yes	Yes	Yes	Yes	Yes	Yes
Year fixed effect	Yes	Yes	Yes	Yes	Yes	Yes	Yes
Enterprise fixed effect	Yes	Yes	Yes	Yes	Yes	Yes	Yes
F value	713.1	8.741	648.3	649.7	11.52	713.1	8.741
*R-squared*	0.681	0.270	0.681	0.681	0.256	0.681	0.270
Obs.	326032	326620	326032	326620	326032	326620	326032

Note: The levels of ***, ** and * are significant at 1%, 5% and 10%, respectively. The model controls the year and enterprise fixed effect, the standard error presented in parenthesis adopted at enterprise level clustering robustness. The joint significance test F value shows that all variables of the model are overall significant. The goodness of fit *R-squared* results show that the models explain the changes of dependent variables to varying degrees, and they are all reasonable for the panel model.

### Discussion on endogeneity

The main independent variable is environmental regulation. As an environmental policy, it is exogenous to some extent; however, environmental regulation intensity is correlated with regional pollution levels and production activities. If the main independent variables are not strictly exogenous, the regression results will be biased and inconsistent.

Prior to application of the Heckman two-step method [44] and 2SLS method, it is necessary to determine the instrumental variables corresponding to the main variables. In the relevant literature, most scholars introduce an endogenous variable lag value of the first order and an average value of the variable as instrumental variables for the main independent variables [65]. In terms of environmental regulation, we follow [66] and introduce the concept of air flow coefficient (found in meteorology) as an instrumental variable. Specifically, based on the grid data ERA-INTERIM published by European Centre for Medium-Range Weather Forecasts, an air flow coefficient index describing air mobility at the prefectural and city levels in China is constructed. The relevant formula is similar to that of [66] and [67]:

$$AIRCUR_{ct}=WINDS_{ct}\cdot BOUNH_{ct}$$ (24)

Where *AIRCUR*_*ct*_ is the air flow coefficient, *WINDS*_*ct*_ is the wind speed of 10 meters per second, and *BOUNH*_*ct*_ is the height of atmospheric mixing layer (or boundary layer). The ArcGIS software is used to analyze the latitude and longitude grid data into the data at the city level. *c* and *t* represent city and year, respectively. Theoretically, when the air pollutant emissions are identical, cities with low air flow coefficient tend to adopt more stringent environmental regulation tools; the instrumental variables have a strong correlation with endogenous variables and a weak correlation with error terms and dependent variables, thus satisfying the conditions for instrumental variables. Therefore, it is reasonable to choose air flow coefficient as the instrumental variable for environmental regulation.

The relevant results are shown in Table 11. Columns (1)-(2), (3)-(4), and (5)-(6) represent sample selection bias test results of the impact of command-control, market-incentive, and voluntary-participation environmental regulation on export technological sophistication, respectively. Columns (1), (4), and (7) and (2), (5), and (8) are Heckman’s regression results in the second stage. From the results, the inverse-Mills ratio is statistically significant in all the regression results, indicating that the model suffers sample selection bias to a certain extent. However, the results were consistent with the benchmark results. Therefore, we suggest that, although there is a certain degree of sample selection bias, the regression results for the main variables did not cause obvious interference.

**Table 11:** Regression results of endogeneity.

Variable	(1)	(2)	(3)	(4)	(5)	(6)	(7)	(8)	(9)
	Heckman Two-step		2SLS	Heckman Two-step		2SLS	Heckman Two-step		2SLS
*OER*	-0.391***		-8.577***						
	(0.05)		(2.92)						
*OERS*	0.293***		5.781**						
	(0.03)		(2.38)						
*MER*				0.123***		2.294**			
				(0.05)		(0.92)			
*MERS*				-0.062**		-0.037*			
				(0.03)		(0.020)			
*PER*							0.293***		0.774***
							(0.06)		(0.19)
*PERS*							-0.086***		-0.166**
							(0.02)		(0.08)
*IMR*		2.056***			4.532***				4.679***
		(0.21)			(0.59)				(0.61)
Constant	13.349***	6.468***	15.006***	9.060***	0.227***	14.454***	8.860***	0.227***	15.560***
	(0.26)	(0.10)	(0.34)	(0.55)	(0.06)	(0.29)	(0.57)	(0.06)	(0.68)
Control var.	Yes	Yes	Yes	Yes	Yes	Yes	Yes	Yes	Yes
U-shaped test			2.32***			3.14			0.58
P value			0.010			0.252			0.281
Reflection point									
rk LM test			105.59			25.36			1066.41
Wald rk F test			36.85			13.60			671.85
Wald test			15.28			23.67			96.78
Year fixed effect	Yes	Yes	Yes	Yes	Yes	Yes	Yes	Yes	Yes
Enterprise fixed effect	Yes	Yes	Yes	Yes	Yes	Yes	Yes	Yes	Yes
Obs.	354953	354953	326032	354953	354953	354953	354953	354953	354953

Note: The levels of ***, ** and * are significant at 1%, 5% and 10%, respectively. The model controls the year and enterprise fixed effect, the standard error presented in parenthesis adopted at enterprise level clustering robustness.

Columns (3), (6), and (9) show the results of the reverse causality endogeneity test of the regression models. The results are consistent with the benchmark results. Moreover, the results of the LM test introduced by [68] have LM test values of 105.59, 25.36 and 1066.41, respectively, indicating that the selected instrumental variable is identifiable. The Wald rk F test are 36.85, 13.60 and 671.85, respectively, also indicating that the selected instrumental variable is highly identifiable. The Wald test values of [69] are 15.28, 23.67 and 97.78, respectively, suggesting that the instrumental variable is not weak. All the above test results suggest that there is no obvious problem with the validity of the instrumental variables.

## Conclusions and implications

We investigated the impact of environmental regulation on the export technological sophistication of manufacturing enterprises, and tested the theoretical mechanism behind the impact. Additionally, we performed an in-depth analysis of the impact on heterogeneity in terms of trade pattern, location, and ownership of enterprises. The relevant research conclusions are as follows.

1. The impact of command-control environmental regulation on export technological sophistication is U-shaped nonlinear, while the impact of market-incentive and voluntary-participation environmental regulation on export technological sophistication is inverted U-shaped nonlinear. Command-control environmental regulation negatively correlates with export technological sophistication, while market-incentive and voluntary-participation environmental regulation positively correlate with export technological sophistication.
2. In terms of heterogeneity of trade patterns, the impact of environmental regulation on the export technological sophistication of enterprises shows nonlinearity. Command-control environmental regulation negatively correlates with the export technological sophistication of mixed trade enterprises. Market-incentive and voluntary-participation environmental regulation positively correlate with the export technological sophistication of enterprises with different trade patterns.
  In terms of heterogeneity of enterprise location, the impact of environmental regulation on export technological sophistication shows nonlinearity. Command-control environmental regulation negatively correlates with the export technological sophistication of enterprises in the eastern regions, while market-incentive and voluntary-participation environmental regulation positively correlate with the export technological sophistication of enterprises in different regions.
  In terms of the heterogeneity of enterprise ownership, the impact of environmental regulation on export technological sophistication shows nonlinearity. Command-control environmental regulation is negatively correlated with the export technological sophistication of foreign-funded enterprises, while market-incentive and voluntary-participation environmental regulation are positively correlated with the export technological sophistication of domestic-funded and foreign-funded enterprises.
3. The mechanism analysis results show that command-control and voluntary-participation environmental regulations have a significant impact on export technological sophistication through green invention and green utility innovation. Market-incentive environmental regulation significantly affects export technological sophistication through green invention, green utility, and green design innovation.

Our conclusions have obvious policy implications. Green invention and green utility innovation are important ways to enhance international competitiveness, and it is fundamental to clarify how environmental regulatory policies may be used to stimulate corporate green innovation enthusiasm and raise green innovation levels. Importantly, relevant departments should treat the use of such policy tools with caution: rigid indicators such as pollutant discharge compliance rate should not be set and used excessively. There is still room for the promotion of market-incentive environmental regulation. The relevant authorities should continue to improve the environmental tax system, such as gradually expanding the tax scope of environmental taxes and fees, strictly controlling tax preferences, etc. In addition, the government should improve the legal basis for emission trading, establish a fair and reasonable initial allocation and pricing mechanism, and accelerate the integration of emission trading and carbon trading, to give full play to its policy tools for the optimal allocation to enterprises. Voluntary-participation environmental regulation is of great significance to the improvement of enterprises’ competitiveness. China should improve the level of enthusiasm of the public to participate more in the construction of environmental protection. The government should clarify the public’s environmental rights by law, enhance the public’s awareness of environmental protection with the help of publicity media, and broaden and enrich the channels and forms of public participation in environmental management.

## References

[1] Bernard AB, Schott RPK. Multiple-product firms and product switching. American Economic Review. 2010;100(1):70–97. 10.1257/aer.100.1.70

[2] Elrod AA, Malik AS. The effect of environmental regulation on plant-level product mix: a study of epa’s cluster rule. Journal of Environmental Economics and Management. 2017;83:164–184. 10.1016/j.jeem.2017.03.002

[3] Dugan MT, Turner EH, Thompson MA, Murray SM. Measuring the financial impact of environmental regulations on the trucking industry. Research in Accounting Regulation. 2017;29(2):152–158. 10.1016/j.racreg.2017.09.007

[4] Lähteenmäki-Uutela A, Repka S, Haukioja T, Pohjola T. How to recognize and measure the economic impacts of environmental regulation: The Sulphur Emission Control Area case. Journal of Cleaner Production. 2017;154:553–565. 10.1016/j.jclepro.2017.03.224

[5] Yang J, Guo H, Liu B, Shi R, Zhang B, Ye W. Environmental regulation and the Pollution Haven Hypothesis: Do environmental regulation measures matter? Journal of Cleaner Production. 2018;202:993–1000. 10.1016/j.jclepro.2018.08.144

[6] De Santis R, Esposito P, Lasinio CJ. Environmental regulation and productivity growth: main policy challenges. International Economics. 2021. 10.1016/j.inteco.2021.01.002

[7] Farzin YH, Kort PM. Pollution abatement investment when environmental regulation is uncertain. Journal of Public Economic Theory. 2000;2(2):183–212. 10.1111/1097-3923.00036

[8] Corsatea TD, Giaccaria S. Market regulation and environmental productivity changes in the electricity and gas sector of 13 observed EU countries. Energy. 2018;164:1286–1297. 10.1016/j.energy.2018.08.145

[9] Wang K, Mi Z, Wei Y-M. Will pollution taxes improve joint ecological and economic efficiency of thermal power industry in china? A DEA-based materials balance approach. Journal of Industrial Ecology. 2019;23(2):389–401. 10.1111/jiec.12740

[10] Blackman A. Can voluntary environmental regulation work in developing countries? Lessons from case studies. Policy Studies Journal. 2008;36(1):119–141. 10.1111/j.1541-0072.2007.00256.x

[11] Jiang Z, Wang Z, Zeng Y. Can voluntary environmental regulation promote corporate technological innovation? Business Strategy and the Environment. 2020;29(2):390–406. 10.1002/bse.2372

[12] Lectard P, Rougier E. Can developing countries gain from defying comparative advantage? Distance to comparative advantage, export diversification and sophistication, and the dynamics of specialization. World Development. 2018;102:90–110. 10.1016/j.worlddev.2017.09.012

[13] Baliamoune-Lutz M. Trade sophistication in developing countries: does export destination matter? Journal of Policy Modeling. 2019;41(1):39–51. 10.1016/j.jpolmod.2018.09.003

[14] Zheng H-H, Wang Z-X. Measurement and comparison of export sophistication of the new energy industry in 30 countries during 2000–2015. Renewable and Sustainable Energy Reviews. 2019;108:140–158. 10.1016/j.rser.2019.03.038

[15] Hausmann R, Hwang J, Rodrik D. What you export matters. Journal of Economic Growth. 2007;12(1):1–25. 10.1007/s10887-006-9009-4

[16] Xu B. The sophistication of exports: Is China special? China Economic Review. 2010;21(3):482–493. 10.1016/j.chieco.2010.04.005

[17] Xu MY, Zuo HP. Research on the relationship between environmental regulation and industrial competitiveness under the agglomeration effect: a retest based on "porter hypothesis". China Industrial Economics. 2013(03):72–84. http://en.cnki.com.cn/Article_en/CJFDTotal-ZGGK201908006.htm.

[18] Xiao XJ, Chen ZP. The effect of environmental regulations on the technical complexity of export commodities and its constraints——based on the threshold regression analysis of my country’s provincial panel data. Collected Essays on Finance and Economics. 2019(10):104–112.

[19] Ladley D. The high frequency trade off between speed and sophistication. Journal of Economic Dynamics and Control. 2020;116:103912. 10.1016/j.jedc.2020.103912

[20] Sandu S, Ciocanel B. Impact of R&D and innovation on high-tech export. Procedia Economics and Finance. 2014;15:80–90. 10.1016/S2212-5671(14)00450-X

[21] Riikonen A, Smura T, Töyli J. The effects of price, popularity, and technological sophistication on mobile handset replacement and unit lifetime. Technological Forecasting and Social Change. 2016;103:313–323. 10.1016/j.techfore.2015.11.017

[22] Salas-Velasco M. Production efficiency measurement and its determinants across OECD countries: The role of business sophistication and innovation. Economic Analysis and Policy. 2018;57:60–73. 10.1016/j.eap.2017.11.003

[23] Wu L, Wei Y, Wang C. Disentangling the effects of business groups in the innovation-export relationship. Research Policy. 2021;50(1):104093. 10.1016/j.respol.2020.104093

[24] Mu M, Zhang Z, Cai X, Tang Q. A water-electricity nexus model to analyze thermoelectricity supply reliability under environmental regulations and economic penalties during drought events. Environmental Modelling & Software. 2020;123:104514. 10.1016/j.envsoft.2019.104514

[25] Schantl SF, Wagenhofer A. Optimal internal control regulation: Standards, penalties, and leniency in enforcement. Journal of Accounting and Public Policy. 2020:106803. 10.1016/j.jaccpubpol.2020.106803

[26] Gowin KD, Wang D, Jory SR, Houmes R, Ngo T. Impact on the firm value of financial institutions from penalties for violating anti-money laundering and economic sanctions regulations. Finance Research Letters. 2020:101675. 10.1016/j.frl.2020.101675

[27] Holland SP. Emissions taxes versus intensity standards: second-best environmental policies with incomplete regulation. Journal of Environmental Economics and Management. 2012;63(3):375–387. 10.1016/j.jeem.2011.12.002

[28] Montero J-P. Permits, standards, and technology innovation. Journal of Environmental Economics and Management. 2002;44(1):23–44. 10.1006/jeem.2001.1194

[29] Yu Y. A study on price discrimination in intermediate goods market from the perspective of antimonopoly—based on monopolistic competition structure and nonlinear pricing in downstream market. East China Economic Management. 2015;1:180–184. https://mall.cnki.net/magazine/Article/HDJJ201501029.htm.

[30] Jiang FX, Wang ZJ, Bai JH. The dual effect of environmental regulation on technological innovation: an empirical study based on dynamic panel data of Jiangsu manufacturing industry. China Industrial Economy. 2013(07):44–55.

[31] Domazlicky BR, Weber WL. Does environmental protection lead to slower productivity growth in the chemical industry? Environmental and Resource Economics. 2004;28(3):301–324. 10.1023/B:EARE.0000031056.93333.3a

[32] Ma Y, Cao H, Ma Y, Wu S. Does technological innovation reduce water pollution intensity in the context of informal environmental regulation? Asia-Pacific Journal of Chemical Engineering. 2020;15(S1):e2493. 10.1002/apj.2493

[33] Hall B. The financing of research and development. Oxford Review of Economic Policy. 2002;18(1):35–51. 10.1093/oxrep/18.1.35

[34] Jaffe AB, Palmer K, Krugman P. Environmental regulation and innovation: a panel data study. Review of Economics and Statistics. 1997;79(4):610–619. 10.1162/003465397557196

[35] Yu MG, Fan R, Zhong HJ. China’s industrial policy and enterprise technological innovation. China Industrial Economy. 2016(12):5–22.

[36] Bu M, Qiao Z, Liu B. Voluntary environmental regulation and firm innovation in China. Economic Modelling. 2020;89:10–18. 10.1016/j.econmod.2019.12.020

[37] Bowen F, Tang S, Panagiotopoulos P. A classification of information-based environmental regulation: Voluntariness, compliance and beyond. Science of The Total Environment. 2020;712:135571. 10.1016/j.scitotenv.2019.135571 31787310

[38] Darnall N. Motivations for participating in a voluntary environmental initiative: the multi-state working group and EPA’s EMS Pilot Program. Elgar Edward: Research in Corporate Sustainability; 2007. 123–154. https://ssrn.com/abstract=1009757.

[39] Lyon TP, Maxwell JW. Self-regulation, taxation and public voluntary environmental agreements. Journal of Public Economics. 2003;87(7):1453–1486. 10.1016/S0047-2727(01)00221-3

[40] Kolcava D, Bernauer T. Greening the economy through voluntary private sector initiatives or government regulation? A public opinion perspective. Environmental Science & Policy. 2021;115:61–70. 10.1016/j.envsci.2020.09.013

[41] Friedman M. The social responsibility of business is to increase its profits. Perspectives in Business Ethics. 1970:225–230. https://hbr.org/2012/04/you-might-disagree-with-milton.

[42] Levinsohn J, Petrin A. Estimating production functions using inputs to control for unobservables. Review of Economic Studies. 2003;70(2):317–341. 10.1111/1467-937X.00246

[43] Wang XL, Yu JW, Fan G. China’s provincial enterprise business environment index 2013 report (Abstracts). Journal of the National School of Administration. 2013(04):24–34.

[44] Heckman JJ. Sample selection bias specification error. Econometrica. 1979;47(1):153–161. http://links.jstor.org/sici?sici=0012-9682%28197901%2947%3A1%3C153%3ASSBAAS%3E2.0.CO%3B2-J.

[45] Sen S. Corporate governance, environmental regulations, and technological change. European Economic Review. 2015;80:36–61. 10.1016/j.euroecorev.2015.08.004

[46] Yu W, Chen Q, Chen H. Environmental regulation, technological innovation and operating performance: based on empirical analysis of 37 industrial industries. Science Research Management. 2017;38(02):18–25.

[47] Wang J, Liu B. Environmental regulation and enterprise total factor productivity: an empirical analysis based on data from Chinese industrial enterprises. China Industrial Economy. 2014(03):44–56.

[48] Zhao XK, Wang YF. Do environmental regulations affect the spatial transfer of pollution-intensive industries?: based on the phased observation of Guangdong. Guangdong Social Sciences. 2016(05):17–32.

[49] Shen J, Wei YD, Yang Z. The impact of environmental regulations on the location of pollution-intensive industries in China. Journal of Cleaner Production. 2017;148:785–794. 10.1016/j.jclepro.2017.02.050

[50] Wang YJ, Shen D, Shi BZ, Li KW. How does infrastructure increase the complexity of export technology? Economic Research. 2010;45(07):103–115.

[51] Kee HL, Tang H. Domestic value added in exports: theory and firm evidence from China. American Economic Review. 2016;106(6):1402–1436. 10.1257/aer.20131687

[52] Zhang J, Chen ZY, Liu YC. The measurement and change mechanism of China’s export domestic value added. Economic Research. 2013;48(10):124–137.

[53] Tian W, Yu MJ. Enterprise export intensity and imported intermediate goods trade liberalization: an empirical study from Chinese enterprises. Management World. 2013(01):28–44. http://ir.calis.edu.cn/hdl/211010/21465.

[54] Ahn JB, Khandelwal AK, Wei SJ. The role of intermediaries in facilitating trade. Journal of International Economics. 2011;84(1):0–85. 10.1016/j.jinteco.2010.12.003

[55] Koopman R, Wang Z, Wei SJ. Estimating domestic content in exports when processing trade is pervasive. Journal of Development Economics. 2012;99(1):178–189. 10.1016/j.jdeveco.2011.12.004

[56] Brandt L, Van Biesebroeck J, Zhang Y. Creative accounting or creative destruction? Firm-level productivity growth in Chinese manufacturing. Journal of Development Economics. 2012;97(2):339–351. 10.1016/j.jdeveco.2011.02.002

[57] Nie HH, Jiang T, Yang RD. The current status and potential problems of the use of China Industrial Enterprise Database. World Economy. 2012;35(05):142–158.

[58] Tong J, Liu W, Xue J. Environmental regulation, factor input structure and industrial transformation and upgrading. Economic Research. 2016;51(07):43–57.

[59] Lind JT, Mehlum H. With or without U? The appropriate test for a U-shaped relationship. Oxford Bulletin of Economics & Statistics. 2007;72(1):109–118.

[60] Baron RM, Kenny DA. The moderator-mediator variable distinction in social psychological research: conceptual, strategic, and statistical considerations. J Pers Soc Psychol. 1986;51(6):1173–1182. 10.1037//0022-3514.51.6.1173 3806354

[61] Judd CM, Kenny DA. Process analysis:estimating mediation in treatment evaluations. Evaluation Review. 1981;5(5):602–619. 10.1177/0193841x8100500502

[62] MacKinnon DP, Lockwood CM, Hoffman JM, West SG, Sheets V. A comparison of methods to test mediation and other intervening variable effects. Psychol Methods. 2002;7(1):83–104. 10.1037/1082-989x.7.1.83 11928892 PMC2819363

[63] Sobel ME. Asymptotic confidence intervals for indirect effects in structural equation models. Sociological methodology. 1982;13:290–312. https://link.springer.com/article/10.3758/BRM.40.3.879.

[64] Zhang J. Research on the inhibition effect of imports on the patent activities of Chinese manufacturing enterprises. China Industrial Economy. 2015(07):68–83.

[65] Yu LH, Lu Y, Lu JY. Deconstruction of the economic system’s impact on the productivity of Chinese enterprises. Economics (Quarterly). 2014;13(01):127–150.

[66] Broner F, Bustos P, Carvalho VM. Sources of comparative advantage in polluting industries. National Bureau of Economic Research Working Paper Series 18337. 2012;No. 18337.

[67] Hering L, Poncet S. Environmental policy and exports: Evidence from Chinese cities. Journal of Environmental Economics and Management. 2014;68(2):296–318. 10.1016/j.jeem.2014.06.005

[68] Kleibergen F, Paap R. Generalized reduced rank tests using the singular value decomposition. Journal of Econometrics. 2006;133(1):97–126. 10.1016/j.jeconom.2005.02.011

[69] Anderson T, Rubin H. The asymptotic properties of estimates of the parameters of a single equation in a complete system of stochastic equations. The Annals of Mathematical Statistics. 1950;20(1):46–63. 10.1214/aoms/1177729752

