# Correction: Environmental regulation, green innovation, and international competitiveness of manufacturing enterprises in China: From the perspective of heterogeneous regulatory tools

**DOI:** 10.1371/journal.pone.0315514

**Published:** 2024-12-05

**Authors:** Yuanhong Hu, Sheng Sun, Yixin Dai

There is an error in affiliation 2 for authors Sheng Sun and Yixin Dai. The correct affiliation 2 is: Research Department, Postal Savings Bank of China Jiangsu Branch.
